# Transactions of the New York State Medical Society, Albany, 1845

**Published:** 1845-07

**Authors:** 


### *Transactions of the New York State Medical Society, Albany*, 1845.

Our brethren of the State of New York enjoy a great advantage over those of our own and most of the other states of this Union, in their regular organization into Societies in every county, and in the representation of these in one great central or State Society—a regular federal and representative system. By this arrangement, many advantages are gained to the profession, and, consequently, to the community. By bringing together gentlemen of the same profession from distant parts, acquaintances are begotten, friendships are formed, comparisons of views on interesting points of practice and general policy occur, and in many ways, we can conceive, benefit is derived. Certainly, union and harmony are promoted among an influential part of the community, whose education, interests, and honour, declare that there ought to be no schisms among them. Nothing so effectually removes unfounded prejudices and abates personal hostility as direct personal communication. The examples and the sentiments, which are presented under favourable circumstances of association, incite to greater respect for the profession and increased zeal in the promotion of all its interests. " As iron sharpeneth iron, so doth the countenance of a man his friend."

The organization of the physicians in our sister state, we presume, grew out of the legislative enactments for the prevention of quackery; but now that all restrictions are removed, and every man is allowed to practice and collect fees, without regard to his qualification—or rather, without any qualification, (except for obtaining fees,)—we are gratified to find that they are determined to continue their associations; satisfied, from past experience, that benefits accrue from such combinations which no Legislature, however disposed, can prevent or take away. The report of the Transactions of the State Society, now before us, is the first that has occurred under the new order of things, and shows very plainly that a spirit exists among the members that requires no legislative dry-nursing for its support.

Beside the usual lists of members and officers of the State and County Societies, resolutions and other proceedings, it contains the annual address of the President for the year, Dr. Joel A. Wing, and of the Presidents of several County Societies, besides some original communications on medical subjects, one of which we have transferred to our *Record.* We find, also, the law of the State, passed May 6th, 1844, repealing former laws relating to the practice of Physic and Surgery. It consists of five sections; the two first of which repeal the previous enactments that prohibited any person from practicing and demanding fees without license. The remaining sections are as follows :

§ 3. No person shall be liable to any criminal prosecution, or to indictment, for practising physic and surgery without license, excepting in cases of mal-practice, or gross ignorance, or immoral conduct in such practice.

§ 4. All and every person, not being a licensed physician, who shall practise or attempt to practise physic or surgery, or who shall prescribe for, or administer medicines or specifics to or for the sick, shall be liable for damages, in cases of mal-practice, as if such person were duly licensed to practice physic or surgery.

§ 5. Any person, not being a licensed physician, who shall practise or profess to practise physic or surgery, or shall prescribe medicines or specifics for the sick, and shall, in any court having cognizance thereof, be convicted of gross ignorance, mal-practice, or immoral conduct, shall be deemed guilty of a misdemeanor, and liable to a fine of not less than fifty dollars, nor not exceeding one thousand dollars, or imprisonment in the county jail not less than one month, or exceeding twelve months, or both, in the discretion of the court.

Although by this legislative act all restrictions are removed, and every one is allowed to engage in the practice of medicine and surgery that may choose to do so, it will be seen, that ignorant and incompetent individuals are liable, so far as the law goes, to be held to a severe accountability. This would be quite sufficient, and in theory is all that can be desired, if it were possible to carry it out fully and fairly in practice. But who is to prosecute? Few disinterested people will be found willing to take the trouble; and if physicians engage in it, the cry of persecution from the knaves who are interested will effectually paralyze their efforts. So that, on the whole, it is likely little good will result from these salutary provisions, and the whole matter must be left to the discretion or the fears of those who have occasion to employ medical aid. Nothing is clearer to our mind than that physicians should be careful not to interfere in a matter in which they are likely to get no thanks, but leave quacks and their employers to their own folly.

